# Supplementary material for: Visual speech enhances phoneme separability in human superior temporal gyrus
Source: bioRxiv. 2026 Sep 12:2026.09.10.750762. Preprint. [Version 1] doi: 10.64898/2026.09.10.750762 (PMC13622663; doi:10.64898/2026.09.10.750762)
Supplement: Supplementary file 1 [file NIHPP2026.09.10.750762V1-supplement-1.pdf]

bioRxiv preprint doi: https://doi.org/10.64898/2026.09.10.750762; this version posted September 12, 2026. The copyright holder for this preprint (which was not certified by peer review) is the author/funder, who has granted bioRxiv a license to display the preprint in perpetuity. It is made available under a CC-BY 4.0 International license.



## Supplementary Materials

### *HGp analyses*

For completeness and comparison to other iEEG research, the same analyses were also ran on HGp. After minimal preprocessing steps (see Methods), HGp (70–150 Hz) was extracted using wavelet decomposition and used to train a separate set of electrode-level SVM decoders to decode word labels of the 16 word stimuli. Following the same inclusion criteria for STG restriction and speech responsiveness as in the ERP analyses, electrodes showing significant word decoding when A and AV trials were combined ($p < .001$) were retained for subsequent statistical and hierarchical analyses. For the A and AV analyses, 81 electrodes met the anatomical criterion, of which 20 also passed the functional selection and were retained for subsequent analyses. For the V analyses, 75 electrodes met the anatomical criterion, with 18 retained after functional selection. The number of analyzed bipolar electrodes per patient ranged from 1 to 6 contacts. For A and AV analyses, patients had a mean of 3.33 analyzed contacts (*std* = 1.97; $n$ = 6); for V analyses, patients had a mean of 3.00 analyzed contacts (*std* = 2.37; $n$ = 5). All electrodes included in HGp analyses were depth electrodes (See Fig. S1A).

Overall, electrode-level SVM decoding accuracy was 0.097 (*std* = 0.026) for AV, 0.098 (*std* = 0.012) for A, and 0.060 (*std* = 0.011) for V. Wilcoxon signed-rank test revealed no significant difference in word-label decoding accuracy between AV and A conditions ($W(6) = 7$, $p = 0.594$, $r = -0.060$). Although linear mixed-effects model showed no significant accuracy difference across representational levels, $F(3, 20) = 1.300$, $p = 0.302$, $R^2_{marginal} = 0.140$, $R^2_{conditional} = 0.140$, for completeness of analyses, we still performed Wilcoxon signed-rank tests for each of the four levels (Fig. S1C-D). A significant enhancement in the AV condition was observed only at the phoneme level (A: 0.080, AV: 0.089; $W(6) = 19$, $p = 0.047$, $r = 0.728$). No significant differences were found at the word level (A: 0.102, AV: 0.094; $W(6) = 7$, $p = 0.594$, $r = -0.060$), feature level

bioRxiv preprint doi: https://doi.org/10.64898/2026.09.10.750762; this version posted September 12, 2026. The copyright holder for this preprint (which was not certified by peer review) is the author/funder, who has granted bioRxiv a license to display the preprint in perpetuity. It is made available under a CC-BY 4.0 International license.



(A: 0.075, AV: 0.073; $W(6) = 3$, $p = 0.953$, $r = -0.642$), or for other error types (A: 0.045, AV: 0.046; $W(6) = 9$, $p = 0.656$, $r = -0.128$).

Similarly, linear mixed-effects model showed no significant confidence difference across representational levels, $F(3, 20) = 1.204$, $p = 0.334$, $R^2_{marginal} = 0.131$, $R^2_{conditional} = 0.131$, and for completeness of analyses, we still performed Wilcoxon signed-rank tests for each of the four levels (Fig. S1E-F). No significant differences were observed at the word level (A: 0.074, AV: 0.073; $W(6) = 15$, $p = 0.219$, $r = 0.385$), phoneme level (A: 0.068, AV: 0.069; $W(6) = 18$, $p = 0.078$, $r = 0.642$), feature level (A: 0.068, AV: 0.067; $W(6) = 12$, $p = 0.422$, $r = 0.128$), or for other error types (A: 0.057, AV: 0.057; $W(6) = 7$, $p = 0.781$, $r = -0.300$).

bioRxiv preprint doi: https://doi.org/10.64898/2026.09.10.750762; this version posted September 12, 2026. The copyright holder for this preprint (which was not certified by peer review) is the author/funder, who has granted bioRxiv a license to display the preprint in perpetuity. It is made available under a CC-BY 4.0 International license.



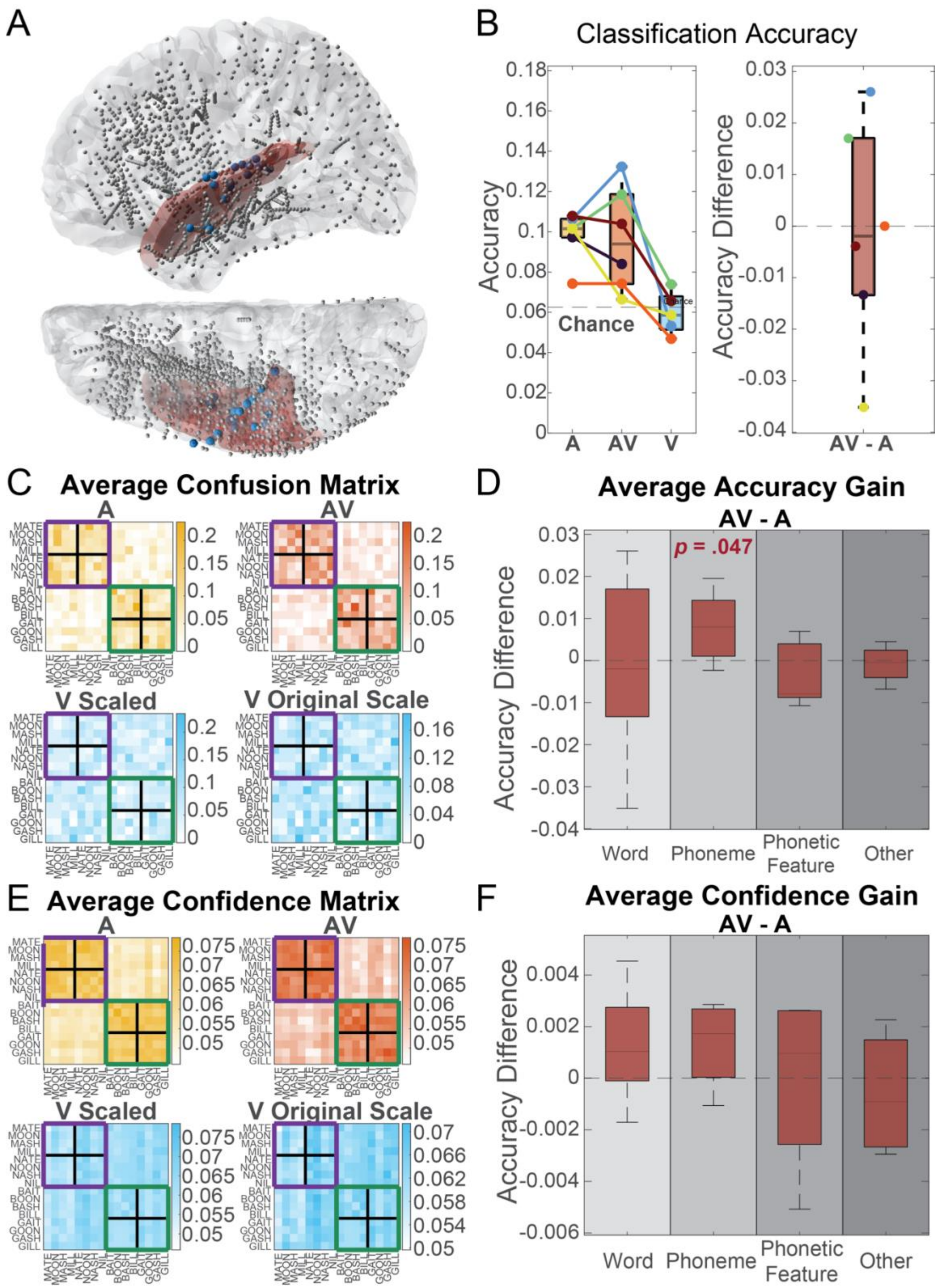


**Figure S1: SVM Decoding for word labels and hierarchical analyses using HGp**

Electrode-level SVM decoding results for 16 word labels using HGp. Six of twelve patients had at least one auditory-responsive electrode showing above-chance decoding performance when A and AV trials were combined. Panels C and D show hierarchical analyses of discrete decoding outcomes from the confusion matrix for each condition, and panels E and F show the corresponding analyses of graded representational shifts based on classifier confidence. The figure layout is identical to that of Figs. 2 and 3. HGp-based analyses showed a similar pattern to the ERP-based results, with phoneme-level, but not feature-level, representations modulated by congruent visual speech.

bioRxiv preprint doi: https://doi.org/10.64898/2026.09.10.750762; this version posted September 12, 2026. The copyright holder for this preprint (which was not certified by peer review) is the author/funder, who has granted bioRxiv a license to display the preprint in perpetuity. It is made available under a CC-BY 4.0 International license.



***Table S1***

*Complete word set in the study*

| | /eɪt/ | /uːn/ | /æʃ/ | /ɪl/ |
|---|---|---|---|---|
| NASAL | **MATE** | **MOON** | **MASH** | **MILL** |
| (/m/, /n/) | **NATE** | **NOON** | **NASH** | **NIL** |
| PLOSIVE | **BAIT** | **BOON** | **BASH** | **BILL** |
| (/b/, /g/) | **GAIT** | **GOON** | **GASH** | **GILL** |

*Note.* Word stimuli used in the current study. Words were selected so that the initial consonants formed two phonetic-feature groups based on manner of articulation: nasals (/m/, /n/) and plosives (/b/, /g/). Within each column, words shared the same vowel nucleus and consonant coda.
